# Stability of oil-in-water emulsions performed by ultrasound power or high-pressure homogenization

**DOI:** 10.1371/journal.pone.0213189

**Published:** 2019-03-08

**Authors:** Yujie Li, Dong Xiang

**Affiliations:** 1 College of Food Science, Hainan University, Haikou, Hainan, China; 2 Engineering Research Center of Utilization of Tropical polysaccharide resources, Ministry of Education, Haikou, Hainan, China; University of New South Wales, AUSTRALIA

## Abstract

Emulsifiers are added to enhance product stability to obtain a satisfactory shelf-life. For this reason, stable emulsions that do not form peroxides nor change the fatty acid composition of food, as well as safe treatments to obtain them, are aspects of utmost importance. High-pressure homogenization is a conventional approach to prepare emulsions because of its high efficiency. In addition, the beneficial effects of ultrasound on the processing efficiency are known. Therefore, the impact of high-pressure homogenization (30 MPa, 50M Pa) or ultrasound power (270 W) on the emulsion stability and emulsifying properties of 5% coconut oil-in-water emulsion were discussed in this study. The complexes (3:7and 4:6, by weight) of propylene glycol alginate and xanthan gum were selected as emulsifier. The apparent viscosity, particle size and distribution, emulsifying properties and ζ–potential of 5% coconut oil-in-water emulsion before and after ultrasound treatment or high-pressure homogenization were investigated and compared. The micro structure of the emulsion was observed under the fluorescence microscope. The experimental results showed that both high-pressure homogenization and ultrasonic treatment effectively reduced the apparent viscosity, average droplet size and narrowed the distribution range of the emulsion, compared with the pre-emulsion. However, aggregation in the emulsion appeared only after being subjected to high-pressure homogenization, while the emulsion made by the ultrasound treatment remained stable during 30 days storage. In conclusion, this study provides valuable information regarding emulsion preparation methods that can be feasible in food and beverage industries, demonstrating a better performance of ultrasound in optimizing and extending food shelf-life in food and beverage industries.

## Introduction

Oil-in-water emulsions are conventionally defined as a thermodynamically unstable systems which include two immiscible liquids (generally water and oil), in which oil is distributed into the water[1]. Emulsions maybe divide into two phases over time through creaming, coalescence, flocculation or Ostwald ripening[2]. The stability of emulsions is the most important parameters for the shelf life of the food products[3]. The preparation method is great influencing on the stability of o/w emulsion. Currently, some ways were used to prepare emulsions such as mechanical, ultrasound (US) treatment and high-pressure homogenization.

Ultrasound(US) power is widely used to a lot of physical and chemical processes such as emulsification, dispersion and chemical reactions[4]. Recently, the attention has been concentrated on the effects of US power on the properties of emulsification and rheological properties of food hydrocolloids [5,6]. US power is an effective and environmentally friendly method to prepare oil-in-water emulsions[4]. Indeed, the shear force generated by the cavitation of US power can disperse oil droplets into small particles with narrow size distribution. So US power can produce o/w emulsions with good stability.

High-pressure homogenization was used in food industry frequently, due to the high efficiency in industrial scale up[7]. High-pressure homogenization is suitable for fluid materials because high pressure homogenization is a continuous non-thermal processing[8]. In this process, fluid materials are subjected to high pressure through the narrow homogenization gap at high speed. The shearing forces from the process of high-pressure homogenization can bring about molecular refinement[9]. Therefore, high-pressure homogenization is often used to produce o/w emulsions.

The main purpose of this paper was to compare the effects of high-pressure homogenization with US on the stability of 5% coconut oil o/w emulsions. Because label-friendly products containing natural and sustainable ingredients are increasingly in demand, polysaccharide-based emulsifiers (complexes of propylene glycol alginate -PGA- and xanthan gum -XG-) were selected. The mass ratio between PGA and XG was determined by measuring the tension on the coconut oil interface. In order to compare the effect of these two approaches on emulsions stability, apparent viscosity, particle size and distribution, and ζ-potential of 5% coconut oil-in-water emulsions prepared by high-pressure homogenization and ultrasound treatment were analyzed and compared. The morphology of oil droplets in emulsions was observed under the fluorescence microscope. Our results provide valuable information for selecting the optimal method in making emulsions feasible for food and beverage industries.

## Materials and methods

### Materials

PGA (>90% purity, 75% degree of esterification) was purchased from Yuanye Chemical Reagent Co., Ltd. XG with an average molecular weight of 4,000 kDa was purchased from Sigma-Aldrich Chemical Reagent Co., Ltd. (St. Louis, MO, USA). Commercial coconut oil was purchased from supermarket (QianCheng Food Company, Hainan, China). All the other chemicals used were of analytical grade and purchased from Sinopharm Chemical Reagent Co., Ltd., China.

### Preparation of the polysaccharides solution

An amounts of the 0.6 g complexes of propylene glycol alginate and xanthan gum were dispersed in 100 ml ultrapure water and then mixed under mild stirring at room temperature (approximately 25 °C). The dispersion was incubated overnight at room temperature to ensure a complete hydration of the complexes. PGA:XG proportions in the mixtures were 3:7, 4:6, 5:5, 6:4 and 7:3 (by weight -wt-), with a total gum concentration of 0.6% (wt).

### Interfacial characteristics measurement

The interfacial tension was measured at the interface of the coconut oil-polysaccharide solution using a drop shape analysis instrument (DropMeter A-60, MAIST, Ningbo, China) at 30 °C, according to a previous report [10]. The helix was twisted to obtain a suitable droplet size in the oil and to measure the falling droplet. Measuring interfacial tension based on the shape of the falling droplet.

### Preparation of o/w emulsions

#### US treatment

**Fig 1:**
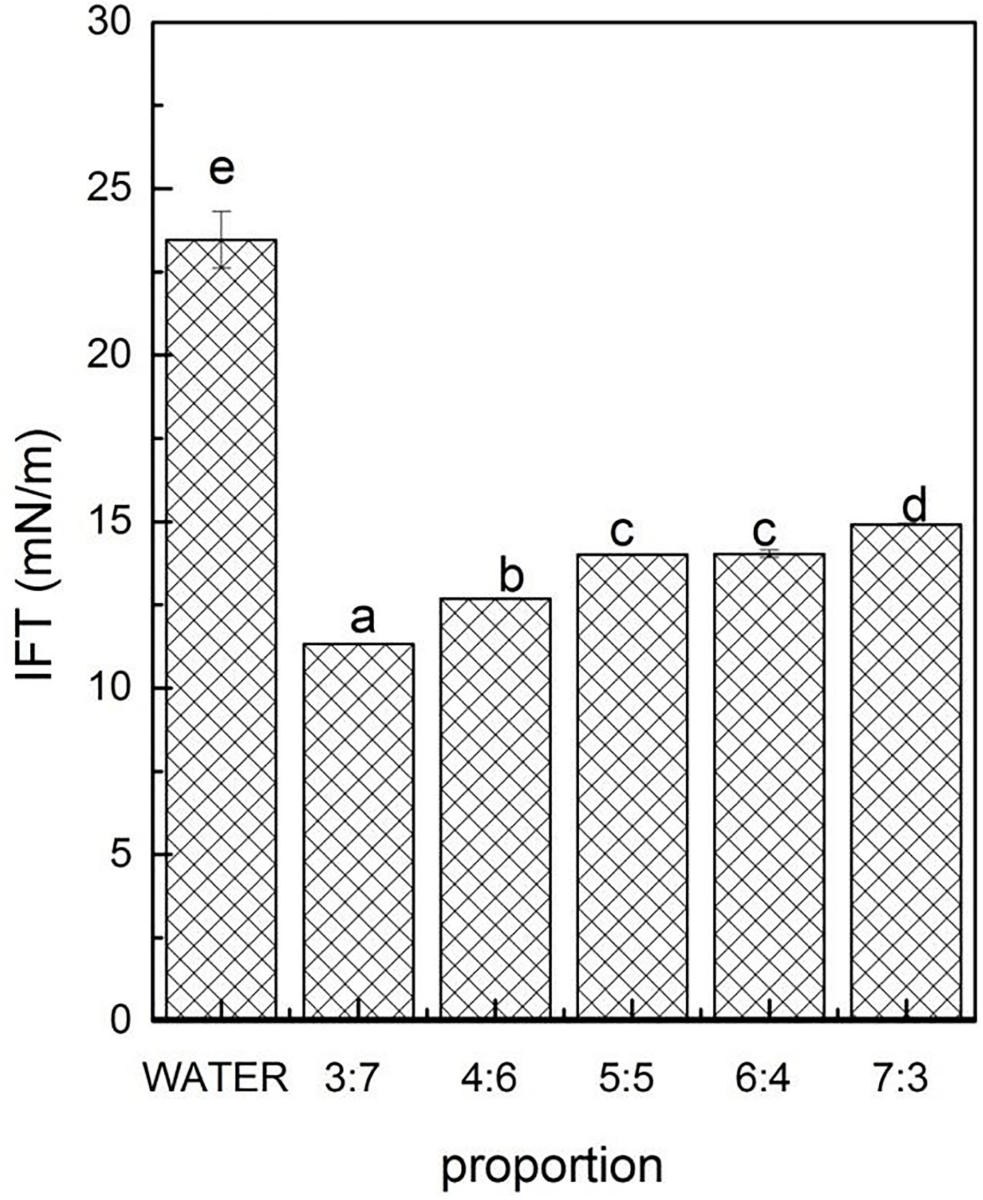
Effect of different proportions of PGA and XG on the interfacial tension measured at the coconut oil-polysaccharide solution interface. Data are expressed as mean ± SD/SEM from three independent replicates (n = 3) for each sample. Sample designated with different lowercase letters(a, b, c and d) indicate significant difference(p<0.05) when compared between different treatment.

Coconut oil (5g) was added into the 95g as-prepared polysaccharide solution (0.6% wt). The mixture was then pre-homogenized using a high shear homogenizer (FJ-200; Biaoben Instruments, Shanghai, China) at a speed of 18000 rpm for 2 min at 30 °C. The resulting coarse emulsion was treated with US (FS-600N, Shangchao, Shanghai, China) (Fig 1) at constant power of 270 W for 7 min according to our previous experiments(data was shown in Supporting information). The coarse emulsion was placed into a 200 ml glassware containing cold water to prevent overheating of the emulsion during US treatment. Finally, 0.005 g sodium azide was added as an antimicrobial preservative.

#### High-pressure homogenization

Coconut oil (5 g) was added into the 95 g as-prepared polysaccharide solution (0.6% wt). The mixture was then pre-homogenized using a high shear homogenizer (FJ-200; Biaoben Instruments, Shanghai, China) at a speed of 18000 rpm for 2 min at 30°C. Then, the primary emulsion was subjected to a cycle of 30 MPa and 50 MPa for three times using a high pressure homogenizer(APV2000, APV Instrument, Germany) to obtain the final emulsion. Finally, 0.005 g sodium azide was added as an antimicrobial preservative.

### Viscosity measurement

The apparent viscosity of the o/w emulsions was measured at 30°Cby a Brookfield viscometer (Massachusetts, USA) using the No. 61 and No. 62 rotors. The polysaccharide solutions before and after US treatment or high-pressure homogenization were separately measured. About 50 ml of the emulsion was placed to the sample celling. The temperature was constant(30 °C) by circulating water from a constant temperature circulartor. The speed of rotor speed was 50r/min.

### Droplet size measurement

Average droplet size and size distribution of o/w emulsions were determined by Laser particle size analyzer (Yidian, WJ-60, Shanghai, China). Exactly 0.5 ml emulsion was added to the sample cell to measure the average particle size and particle size distribution. The average particle size was calculated and the particle size distribution curve was obtained through the software match with the instrument.

### Optical microscopy

Emulsions were observed under a light microscope equipped with a digital camera(cannon, Japan). Images were analyzed using a specific software program. About 0.01ml emulsion samples before and after US treatment or high-pressure homogenization stabilized for 1 day was placed on a glass slide and covered with a coverslip. After 30 min of equilibration, samples were examined at room temperature at 100x magnification.

### Fluorescent microscopy

A fluorescence microscope (Leica DMI DM6000B, Leica Microsystems, Heidelberg, Germany) was applyed to visualize the microstructure of o/w emulsions. This microscope operated in fluorescence mode, with 10x objective of numerical aperture 0.40. The oil droplets were stained with Nile red fluorescent dye. Images were analyzed using a specific software program. An aliquot of emulsion samples before and after US treatment or high-pressure homogenization stabilized for 1 day was placed on a microscope slide. A cover slip was placed on top of the microscope slide, being careful to not trap air bubbles. The samples were observed at 30 °C.

### Emulsification characteristics

The emulsion prepared by US treatment or high-pressure homogenization was diluted 500-fold with 0.05% sodium dodecyl sulfate (SDS) and the absorbance (A) of the diluted emulsion was measured using a spectrophotometer at a wavelength of 500 nm. The emulsification activity index (EAI, m^2^/g) was calculated according to the following formula:

$$EAI=\frac{2.303\times 2\times A\times N}{C\times \emptyset \times 10000}$$

A- absorbance at 500 nm;

N- emulsion dilution factor (500);

C- emulsifier concentration (0.6%);

∅-volume fraction of the oil phase (5%)

The absorbance at the beginning (A0) and the absorbance of the diluted emulsion after standing for 10 minutes were measured by a spectrophotometer at a wavelength of 500 nm (A10). The emulsion stability index (ESI) was calculated according to the following formula:

$$ESI=\frac{A0}{A0-A10}\times 100\%$$

### Zeta potential measurement

The Zeta potential (ζ) of the particles in the oil-in-water emulsions was determined by particle electrophoresis (Zetasizer Nano ZS-90, Malvern Instruments, Worcestershire, UK). To avoid multiple scattering effects, emulsions were diluted 100-fold with buffer solutions of the same pH and ionic composition prior to measurement.

### Statistical analysis

Statistical analysis was performed used the Statistical Analysis Systems (Origin 9.0) software package. All measurements were performed in triplicate and results were expressed as mean ± standard deviation (SD). One-way analysis of variance (ANOVA) was performed to compare different groups and a value of p < 0.05 was considered statistically significant.

## Results and discussion

### Interfacial characteristics of polysaccharide solutions

The interfacial characteristics of surfactants are significant factor in determining their ability to form and stabilize emulsions[11]. In this study, the interfacial tension between different mass ratio of polysaccharide solutions and coconut oil were measured (Fig 1). The experimental results indicated that PGA-XG complexes significantly reduced the interfacial tension compared with water (23.46 mN/m) at oil-water interfaces, because PGA combination with XG resulted in a mixture containing both hydrophilic bonds and hydrophobic bonds, which can be effectively adsorbed at the interface of oil-water. Different mass ratios of PGA and XG resulted in different ability to reduce the interfacial tension. According to result of Fig 1, the mass ratio of 3:7 and 4:6 (PGA: XG, wt) can effectively reduce the interfacial tension to 11.32 mN/m and 12.68 mN/m, which were smaller than other mass ratio. Therefore, 3:7 and 4:6 mass ratios were selected as the emulsifier in this study.

### Viscosity measurement

**Fig 2:**
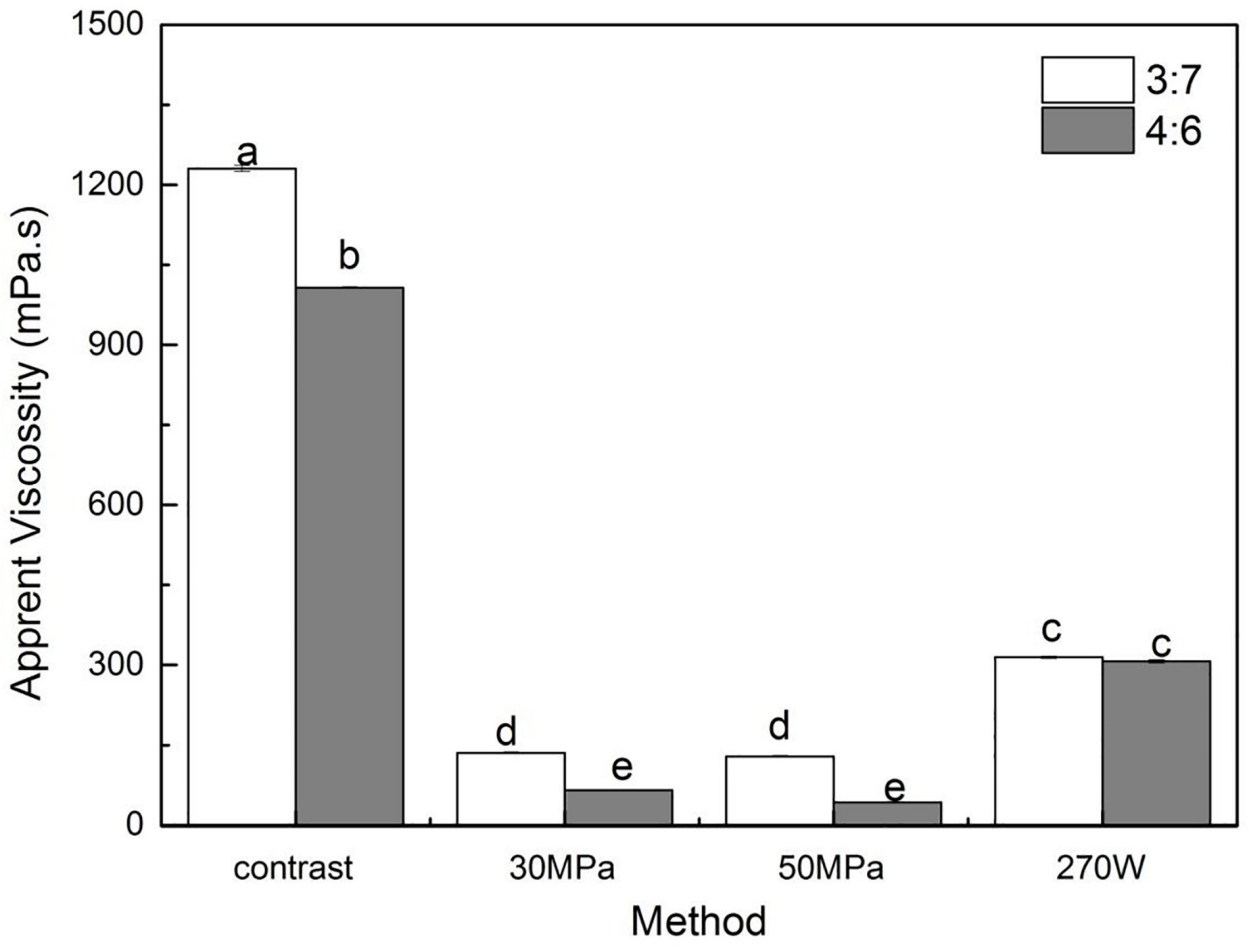
Effect of high pressure homogenization (30 MPa and 50 MPa) and US (270 W) treatment on the apparent viscosity of o/w emulsion. Data are expressed as mean ± SD/SEM from three independent replicates (n = 3) for each sample. Sample designated with different lowercase letters(a, b, c, d and e) indicate significant difference(p<0.05) when compared between different treatment.

The apparent viscosity of oil-in-water emulsions is important for certain applications in food industry [12,13]. Therefore, apparent viscosity of o/w emulsions before and after US treatment or high-pressure homogenization were investigated (Fig 2). The apparent viscosity of 5% coconut oil-in-water pre-emulsions stabilized by a PGA-XG 3:7 mass ratio was 1233.3 mPa.s, which is higher than that stabilized by the 4:6 mass ratio(1006 mPa.s), indicating that XG had an effect on the apparent viscosity of the oil-in-water emulsion. In the previous research, XG acts as a stabilizer in the o/w emulsion[14]. Hydrogen bond can be formed between XG molecules and water molecules. The gel network structure can prevent oil droplets aggregation that affects the stability of oil-in-water emulsions[15,16].

High-pressure homogenization and ultrasound treatment significantly reduced the apparent viscosity of the o/w emulsions, compared to the pre-emulsions (Fig 2). The apparent viscosity of the o/w emulsion prepared by ultrasonic treatment at 270 W (316 mPa.s)was higher than that of the emulsion prepared by high pressure homogenization at 30 MPa(136.4 mPa.s) and 50 MPa (127 mPa.s)homogenization pressure. The changes in apparent viscosity can reflect the changes in molecular weight of polysaccharide[17]. Thus, the experimental results showed that the US wave and high-pressure homogenization treatment could degrade macromolecular polymers to reduce their molecular weight[5,18].

### Particle size and size distribution of fresh emulsions

**Fig 3:**
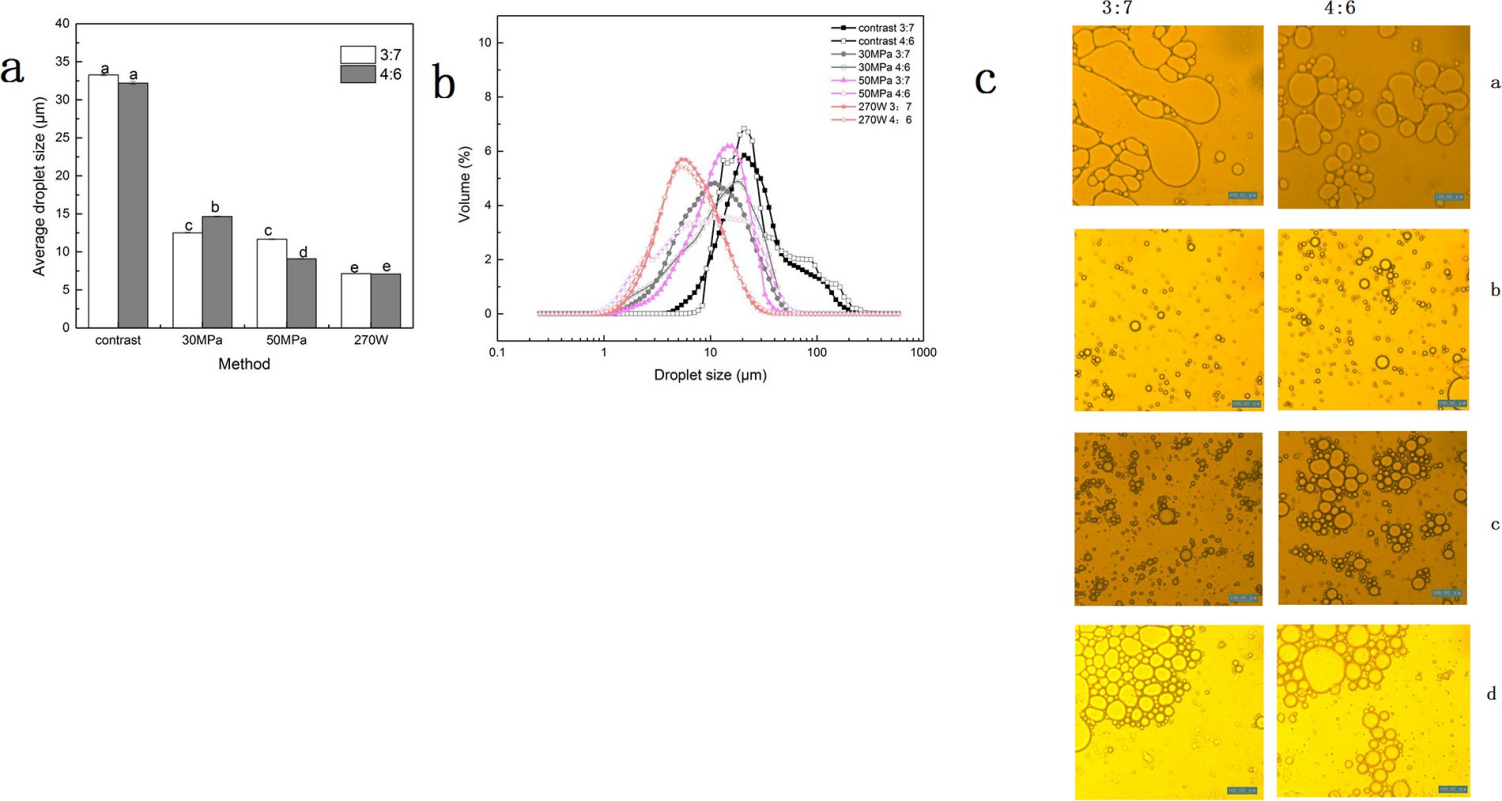
Average article size and particle size distribution of oil-in-water emulsions prepared by different methods. (a) Average droplet size of o/w emulsions prepared by different methods, the pre-emulsion as a comparison. (b) Particle size distribution of 5% coconut oil o/w emulsions prepared by different methods. The contrast indicates the pre-emulsion. (c) Particle suspensions microstructure in fresh 5% coconut oil o/w emulsions prepared by different methods. 100× magnification, 30 °C temperature(a. pre-emulsions, b. 270 W US treatment, c. 30 MPa high-pressure homogenization, d. 50 MPa high-pressure homogenization). Data are expressed as mean ± SD/SEM from three independent replicates (n = 3) for each sample. sample designated with different lowercase letters(a, b, c and d) indicate significant difference(p<0.05) when compared between different treatment.

Microscopic observation and particle size measurement were used to characterize the stability of the oil-in-water emulsions before and after US treatment or high-pressure homogenization. The average droplet size and distribution of all 5% coconut oil-in-water emulsions are shown in Fig 3. The mechanical energy of ultrasonic waves(7.14μm) and high-pressure homogenization(12.51μm) reduced the average droplet size of o/w emulsions, compared with that of contrast pre-emulsions(33.27μm) (Fig 3(a)). The cavitation of US wave can break the oil droplets into small particle sizes, then the complexes of PGA-XG surrounded the small oil droplet to maintain a small size. During the process of high pressure homogenization, pre-emulsions passed through a narrow gap at high speed. The shearing forces from the process of high-pressure homogenization can bring about molecular refinement [7]. The total concentration of polysaccharide was 0.6%, which could completely cover the droplets surface, which can increase steric repulsion and reduces the van der Waals attraction between the droplets in the oil-in-water emulsion [19]. The average particle size of the emulsion produced under higher pressure (50 MPa) was 11.633μm which was smaller than the average particle size (12.51μm) under lower pressure(30 MPa) (Fig 3(a)). The particle size of the o/w emulsions prepared by US treatment was smaller than that of the emulsions produced by high-pressure homogenization. Aggregation appeared in the 5% coconut o/w emulsions prepared by high-pressure homogenization, which might be related to the low viscosity of the o/w emulsions (Fig 3(a)) [20].

Fig 3(b) shows the droplets size distribution of o/w emulsions. The pre-emulsions showed the widest size distribution, which exhibited the poorest stability (Fig 3(b)). The image observed under the microscope (Fig 3(c)) was consistent with the results obtained regarding the particle size distribution. According to the image from microscope, the particle size of o/w emulsions produced by ultrasonic wave was small and uniform. The particle size of the emulsion prepared by high-pressure homogenization was large and droplet aggregation appeared. This may be related to that droplet breakage and aggregation occurred simultaneously during the process of high-pressure homogenization[3,8,9].

### Droplet microstructure

**Fig 4:**
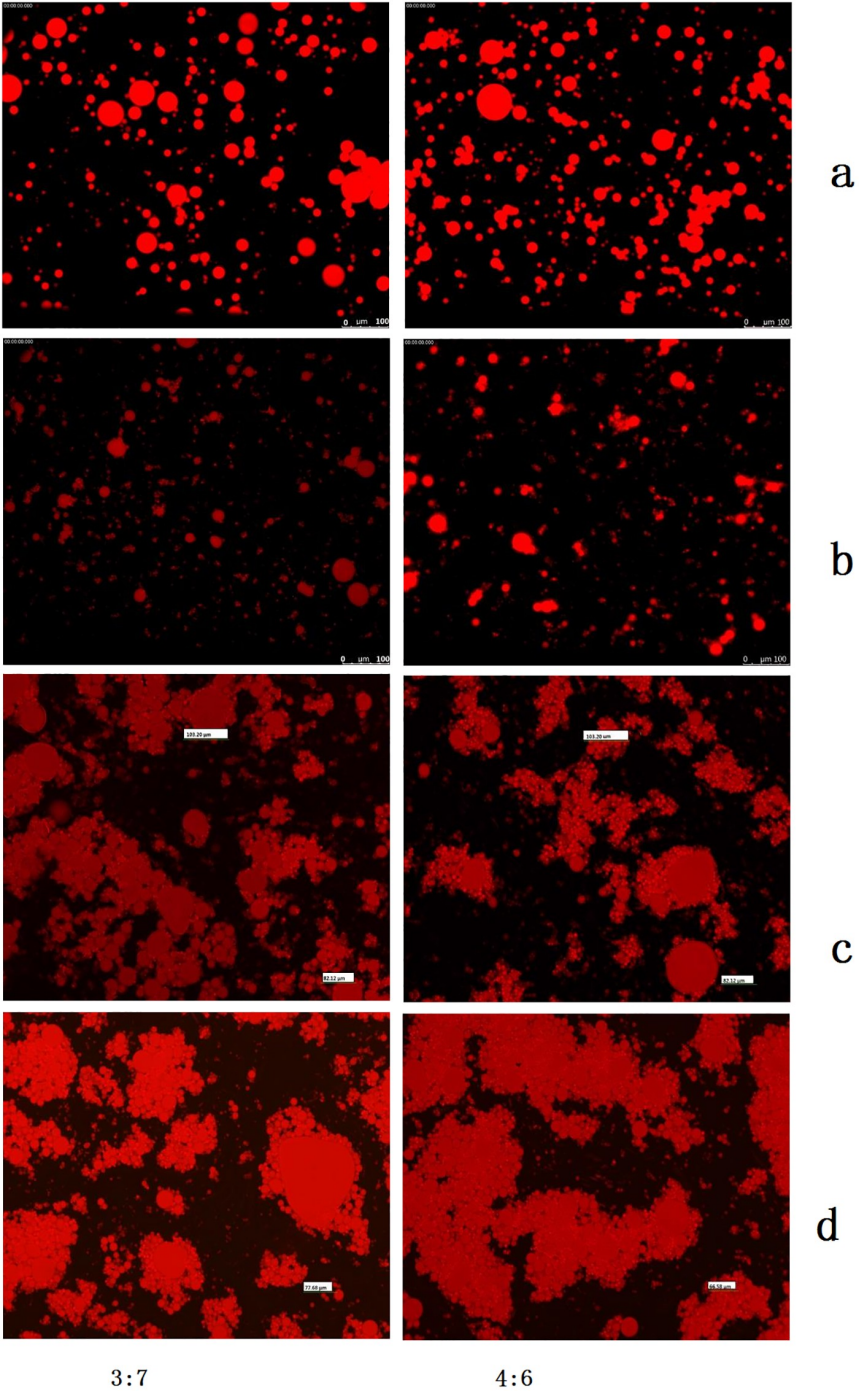
Fluorescent microscopy images of coconut o/w emulsions before and after high-pressure homogenization and ultrasound treatment. a. pre-emulsions, b. 270 W US treatment, c. 30MPa high-pressure homogenization, d. 50MPa high-pressure homogenization.

The microstructure of the emulsions prepared by high-pressure homogenization or ultrasound power is shown in Fig 4. In the pre-emulsions, little or no flocculation was observed, which probably due to the high apparent viscosity, which immobilized the oil droplets, preventing aggregation [21]. The emulsion prepared by 270 W ultrasound treatment showed little or no aggregation. The average particle size of the oil-in-water emulsion treated by US was significantly smaller than that of the pre-emulsion. The treatment of high-pressure homogenization could also reduce the particle size of the oil droplets, but large-scale aggregation occurred in the oil-in-water emulsions and small droplets aggregated together to form large oil droplets. The aggregation in the o/w emulsions obtained under 50 MPa high pressure homogenization was more than that in the emulsions obtained under 30 MPa high pressure homogenization, which might be related to the low viscosity of the emulsions prepared by 50 MPa high-pressure homogenization[18]. Fluorescent microscopy results showed that the stability of the emulsion prepared by 270 W ultrasound treatment was better than that of the emulsion prepared by 30 MPa and 50 MPa high pressure homogenization.

### Emulsifying properties

**Fig 5:**
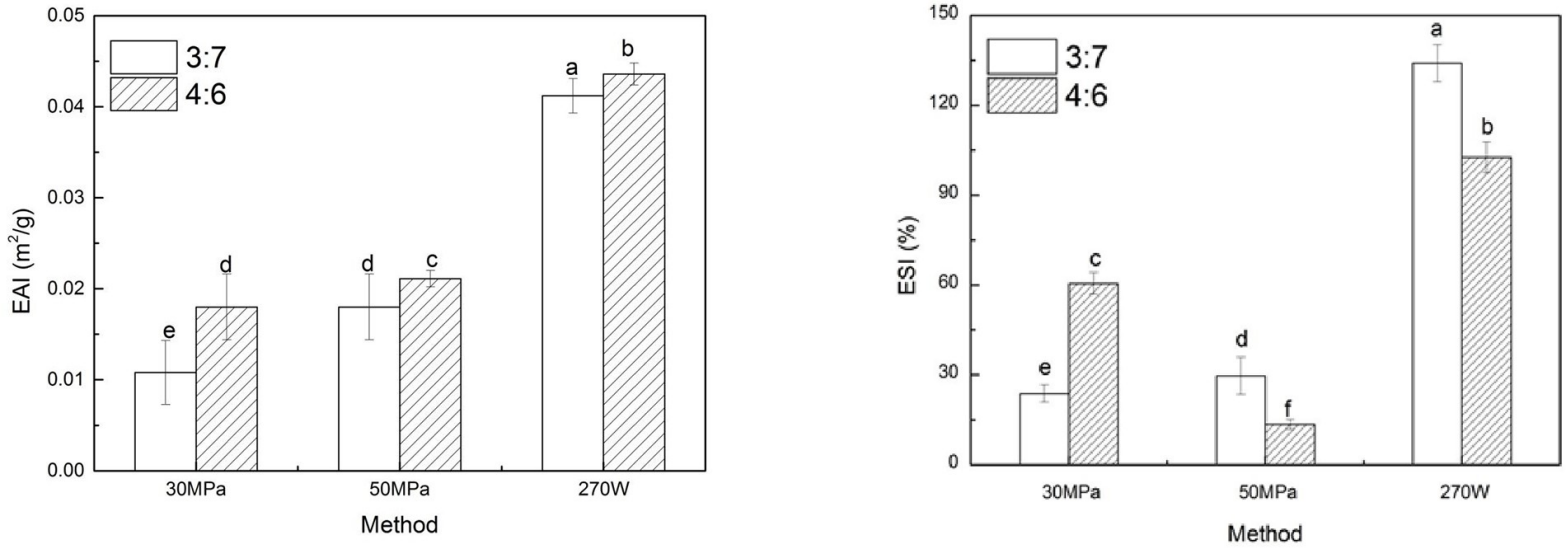
EAI and ESI values of the o/w emulsions prepared by ultrasound treatment or high-pressure homogenization. Data are expressed as mean ± SD/SEM from three independent replicates (n = 3) for each sample. sample designated with different lowercase letters(a, b, c, d and e) indicate significant difference(p<0.05) when compared between different treatment.

Emulsification activity index (EAI) and emulsion stability index (ESI) can reflect the ability of PGA-XG complexes to adsorb at the interface and resist to instability of o/w emulsions such as creaming, coalescence or flocculation[22,23]. The results of EAI and ESI are shown in Fig 5. The EAI of the o/w emulsions prepared by 270 W ultrasound treatment (0.0412 m^2^/g) was higher than that of the o/w emulsions made by 30 MPa and 50MPa high-pressure homogenization(0.0108 m^2^/g) and the pressure had less impact on EAI. The emulsifying properties of emulsifiers are related the surface hydrophobicity which connect with the ability of emulsifier to absorb at oil/water interface[24,25]. Therefore, the degraded polysaccharide chain by 270W ultrasound power can exhibit more hydrophobic bonds and enhance the hydrophobic properties of the PGA-XG complexes. The hydrophobic nature of propylene glycol group in the PGA molecule has interfacial activity, which is capable of blistering, emulsification[2,14]. Therefore, the amount of PGA in the o/w emulsions affects the hydrophobicity of the mixture of PGA-XG. When the amount of PGA is high, the EAI of the emulsion is relatively high.

In order to test the stability of prepared emulsions, the analyses were conducted and results are shown in Fig 5. The highest ESI value(134.083) was observed in the emulsions stabilized by the 3:7 complex of PGA-XG and prepared by 270 W ultrasound treatment. The ESI values of the o/w emulsion represent the measure of the resistance against the coalescence[26]. Thus, emulsions prepared by high-pressure homogenization are more susceptible to coalescence. From the experimental results, the o/w emulsions prepared by 270 W ultrasound treatment presented longer shelf-life.

### ζ-potential measurements

**Fig 6:**
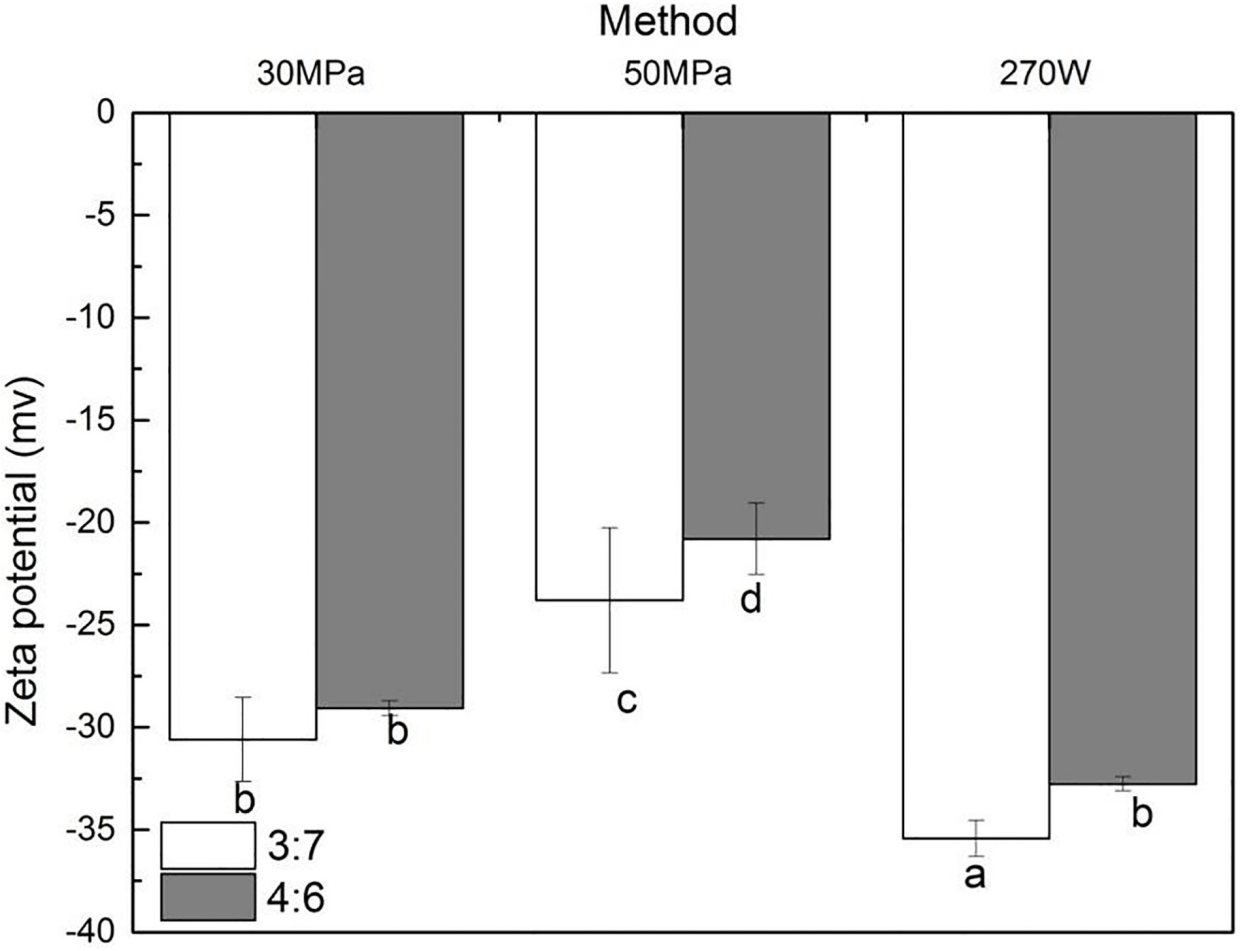
Zeta (ζ) potential of the o/w emulsion samples prepared by different methods. Data are expressed as mean ± SD/SEM from three independent replicates (n = 3) for each sample. sample designated with different lowercase letters(a, b, c, d and e) indicate significant difference(p<0.05) when compared between different treatment.

ζ-potential reflects the charged state of the droplet surface in the o/w emulsion, and it indicates the strength of the repulsion force between droplets. Thus, ζ-potential is an important parameter to assess o/w emulsions stability. Oil-in-water Emulsions with low ζ-potential (negative or positive) are prone to flocculate or coagulate, while o/w emulsions with high ζ-potential are electrically stabilized. The ζ-potential of 5% coconut oil o/w emulsions prepared at 30°C by 270W ultrasound treatment or high-pressure homogenization are shown in Fig 6. XG is known to be a kind of negatively charged hydrocolloid [27]. Sufficient charges can increase the electrostatic repulsion between droplets, which in turn would increase and promote the stability of the oil-in-water emulsions [28]. Our results showed that the ζ-potential of the 270W US-treated emulsion was higher (-36.2mV)than the one of the o/w emulsion prepared by high-pressure homogenization. Julio et al. claimed the existence of a strong connection between ζ-potential and average droplet sizes[29]. When the obtained results were examined in detail, it appeared that the US treatment decreased significantly the average droplet size and polydispersity of the emulsion. The average droplet size of o/w emulsions is shown in Fig 3A and 3B, showing the average distribution of the droplet in the samples: US treatment formed smaller droplets.

### Visual phase separation

**Fig 7:**
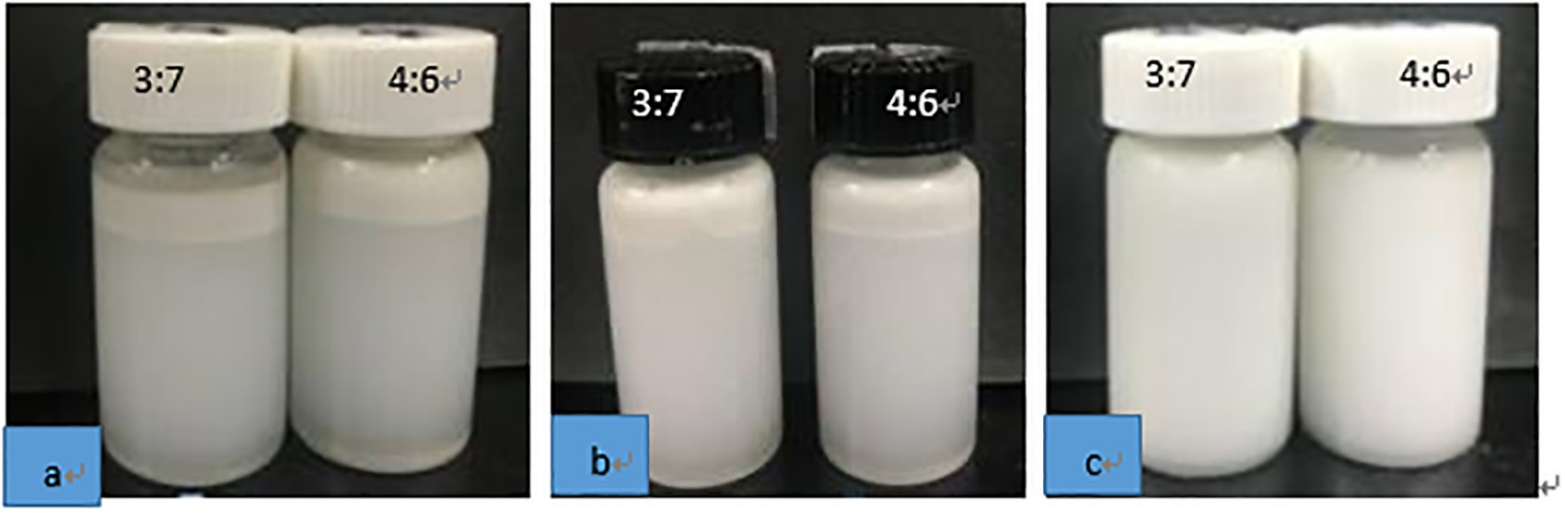
Photographs of 5% coconut o/w emulsions made by different methods. Emulsions were stored at room temperature for 30 days. a, high-pressure homogenization treatment, 30MPa; b, high-pressure homogenization treatment, 50MPa; c, US treatment, ultrasonic power of 270 W.

The appearance of o/w emulsions after storage at 30°C for 30 days is shown in Fig 7. No phase separation was observed in the emulsions prepared by 270W ultrasound treatment (Fig 7c). After 30 days, a partial phase separation was seen in the emulsion prepared by high-pressure homogenization at 30MPa and 50MPa (Fig 7a and 7b). After 30 days storage, the emulsions obtained by US did not appear creamy and maintained a good stability, compared to the emulsions produced by high pressure homogenization.

## Conclusions

High-pressure homogenization and US treatment are two commonly used emulsification method[8,17]. In the previous experiment, the average droplet size of 5% coconut oil-in-water emulsions prepared by 270W ultrasound treatment was smallest compared to that prepared by other ultrasound intensities.(S1 and S2 Figs). In the storage process, the oil-in-water emulsion prepared by 270W ultrasonic treatment did not show phase separation and maintained good stability.(S3 Fig) In this study, comparing the effects of 270W ultrasound treatment on the stability and emulsifying properties of 5% coconut oil-in-water emulsions with 30MPa and 50MPa high-pressure homogenization. The results showed that the pre-emulsions after US treatment and high-pressure homogenization had a larger reduction in apparent viscosity, which consistent with Zhang et [9,30,31]. The average particle size of o/w emulsions prepared by high-pressure homogenization and 270W US treatment were smaller compared to pre-emulsions. The aggregation occurred in the o/w emulsions prepared by 30MPa and 50MPa high-pressure homogenization, resulting in larger average particle size and a wide particle size distribution of the o/w emulsions during storage time. The hydrophobicity of the polysaccharide treated by 270W ultrasound treatment enhanced. so the complexes of PGA-XG treated by 270W US treatment can form relatively thick hydrophilic coating around the coconut oil droplets which can generate strong steric repulsion. This result is consistent with Matos conclusion[32].

Therefore, 270W US treatment could enhance the stability of oil-in-water emulsion systems compared with high-pressure homogenization, which may be due to the fact that the complexes of PGA-XG can quickly adsorb around the broken coconut oil droplets during the process of ultrasonic treatment to generate steric repulsive force to prevent van der Waals attraction, compared with the process of high-pressure homogenization. US treatment can be considered as a safe method to prepare stable o/w emulsions, since it does not form peroxides nor changes in fatty acid composition[26]. As such, future studies should focus on the effect of US on the rheological properties of the o/w emulsions.

## Supporting information

S1 Fig Microstructure of particle suspensions in 5% coconut oil o/w emulsions stabilized by complex PGA and XA prepared by different ultrasound intensities. (TIF)

S2 Fig Average droplet size of 5% coconut oil-in-water emulsions stabilized by different proportions of propylene glycol alginate (PGA) and xanthan gum (XA). Data are expressed as mean ± SD/SEM from three independent replicates (n = 3) for each sample. sample designated with different lowercase letters(a, b, c, d, e and f) indicate significant difference(p<0.05) when compared between different treatment.
(TIF)

S3 Fig General appearance of 5% coconut oil o/w emulsions stabilized by complex of PGA-XG prepared by different ultrasound intensities after 3 days storage at 30 °C. (TIF)
