# Supplementary figures and images for: Stability of oil-in-water emulsions performed by ultrasound power or high-pressure homogenization
Source: PLoS One. 2019 Mar 8;14(3):e0213189. doi: 10.1371/journal.pone.0213189 (PMC6407764; doi:10.1371/journal.pone.0213189)

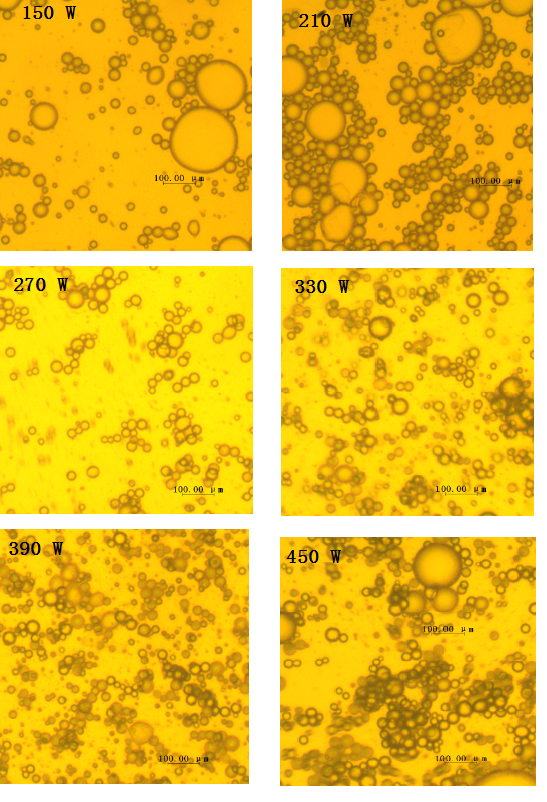

Supplement: S1 Fig — (TIF) [file pone.0213189.s001.tif]

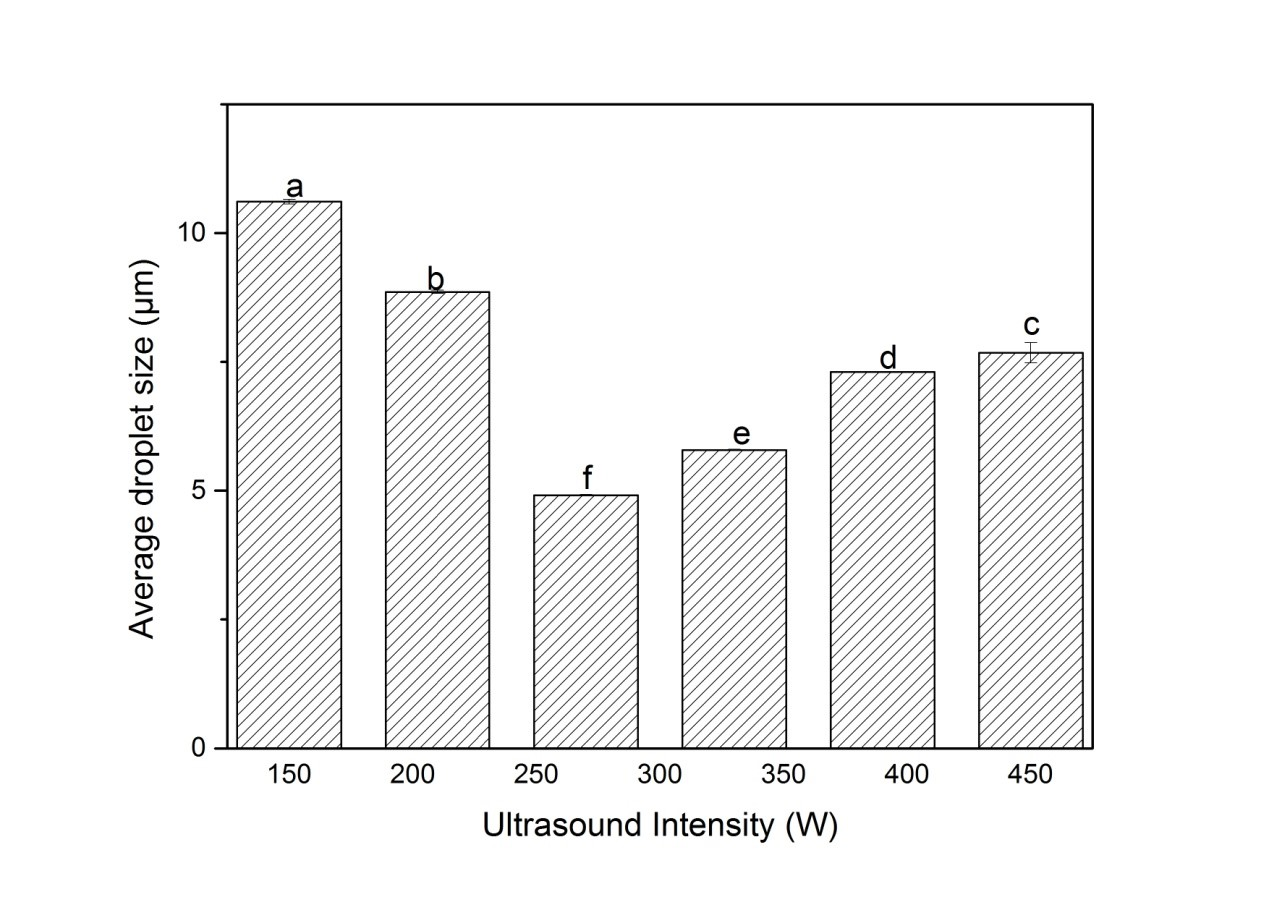

Supplement: S2 Fig — Data are expressed as mean ± SD/SEM from three independent replicates (n = 3) for each sample. sample designated with different lowercase letters(a, b, c, d, e and f) indicate significant difference(p<0.05) when compared between different treatment. (TIF) [file pone.0213189.s002.tif]

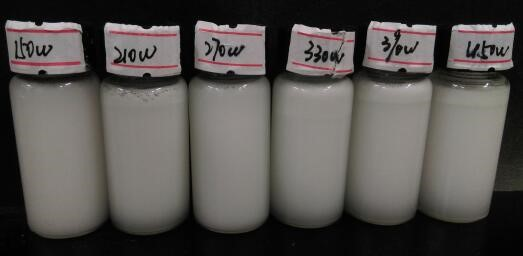

Supplement: S3 Fig — (TIF) [file pone.0213189.s003.tif]
